# Concentrated growth factor inhibits UVA-induced photoaging in human dermal fibroblasts via the MAPK/AP-1 pathway

**DOI:** 10.1042/BSR20193566

**Published:** 2020-07-17

**Authors:** Meng Zhang, Tai Zhang, Yanan Tang, Guiyun Ren, Yanning Zhang, Xiangyu Ren

**Affiliations:** 1 Department of Oral and Maxillofacial Surgery, Stomatological Hospital of Hebei Medical University, The Key Laboratory of Stomatology, Shijiazhuang, Hebei, P.R. China; 2 Department of Jitang college, North China University of Science and Technology, Tangshan, Hebei, P.R. China

**Keywords:** CGF, oxidative stress, photoaging, reversine, UVA-radiation

## Abstract

Ultraviolet (UV) radiation-induced photoaging is one of the contributors to skin aging. UV light triggers oxidative stress, producing a large number of matrix metalloproteinases (MMPs) and degrading the extracellular matrix in skin cells, thereby causing a series of photoaging symptoms. Concentrated growth factor (CGF) is a leukocyte- and platelet-rich fibrin biomaterial that plays a protective role in the occurrence and development of skin photoaging. In the present study, we investigated the underlying mechanism of CGF in the UVA-induced photoaging of human dermal fibroblasts (HDFs). A primary culture of HDFs was isolated from normal human facial skin. The cells were treated with CGF following UVA radiation. Proliferation of cells was detected using MTT assay, followed by measurement of reactive oxygen species (ROS) using immunofluorescence assay and flow cytometry. The mRNA and protein expression levels of P38, c-Jun, and MMP-1 were detected using real-time polymerase chain reaction and Western blot, respectively. CGF was found to improve cell viability by inhibiting the production of ROS and reducing oxidative damage. In addition, there was lower expression of p38 and c-Jun at the mRNA and protein levels following CGF treatment, thus resulting in the inhibition of MMP-1 expression. Our results suggest that CGF could protect HDFs against UVA-induced photoaging by blocking the P38 mitogen-activated protein kinase/activated protein-1 (P38MAPK/AP-1) signaling pathway. These findings provide a new clinical strategy for the prevention of skin photoaging.

## Introduction

Skin is the barrier organ for the reflection and absorption of ultraviolet (UV) rays from the sun. Long-term UV radiation can cause damage to the skin, has a deleterious effect on the skin structure, and induces the symptoms of photoaging [1]. UV light contains three wavebands, long-wave UVA (315–400 nm), medium-wave UVB (280–315 nm), and short-wave UVC (100–280 nm). The atmospheric stratospheric ozone absorbs all of UVC and a small portion of UVB, but not UVA [2]. Skin is composed of two layers—epidermis and dermis. UVB mainly acts on the epidermis and superficial dermis, while UVA can penetrate the basal layer and damage the whole dermis. Therefore, UVA is considered the main culprit responsible for photoaging [3,4].

Skin photoaging is a disease involving the skin and its supporting systems [5]. The connective tissue in the dermis contains collagen in abundance and plays a supporting role in the skin. It has been reported that UVA radiation promotes the production of collagen-degrading enzymes, such as matrix metalloproteinases (MMPs), which leads to imbalance and degradation of collagen synthesis, thereby resulting in skin collapse and wrinkles [6]. MMPs belong to the matrixin subfamily of the large metalloproteinase family and function at natural pH. They are zinc-dependent endopeptidases and are expressed in many different cell types [7]. MMP-1, a subclass of the MMP family, is a collagenase secreted by the fibroblasts in the dermis and is one of the most important factors that affect photoaging [8].

In addition, reactive oxygen species (ROS) are considered as key factors that initiate the signaling pathway of photoaging. Excessive ROS participates in the photoaging process by directly damaging either collagen or the inhibitors of metalloproteinases [9]. The mitogen-activated protein kinase (MAPK) signaling pathway has been well studied in connection to the cellular signaling of photoaging [10]. Extracellular signal-regulated kinase (ERK), p38, c-Jun N-terminal protein kinase (JNK), and ERK5/BMK1 are vital members of the MAPK family. Among them, P38 is vital in regulating the cellular response to UV radiation [11]. UV-induced ROS triggers the cascade reaction of the P38 signaling pathway, following which AP-1 is activated [12]. AP-1, a heterodimer composed of c-Fos and c-Jun, is a transcriptional activator in cells and induces the production of MMPs [13]. Therefore, scavenging ROS, inhibiting the activation of P38 mitogen-activated protein kinase/activated protein-1 (P38MAPK/AP-1) signaling pathway, and reducing the production of MMPs are key strategies for the prevention of UVA-induced photoaging.

Concentrated growth factor (CGF) is a third-generation platelet concentrate product proposed by Sacco in 2006 [14]. It is a novel biomaterial in the field of regenerative medicine that is used for tissue regeneration and differentiation. CGF liquid is obtained from autologous venous blood using differential centrifugation, a process that causes the release of various growth factors. CGF can promote cell proliferation and differentiation, enhance angiogenesis, accelerate the repair of soft and hard tissues, promote skin metabolism, and provide new cells for the damaged skin tissues [15–18]. However, few studies have focused on the effects of CGF in repairing skin photoaging. In our previous study, we showed that CGF is capable of inhibiting UVA-induced photoaging of human dermal fibroblasts (HDFs) [19]; in the present study, we further elucidate the underlying mechanism for this CGF-mediated inhibition of UVA irradiation-induced aging.

## Materials and methods

### Primary culture of HDFs

The present study was approved by the Ethics Committee of the Hospital of Stomatology, Hebei Medical University. The present study was carried out in accordance with the World Medical Association Declaration of Helsinki. Informed consent was obtained from all the participants before the study. Normal human facial skin tissues were obtained from six adults, aged between 19 and 35 years, who underwent a double eyelid surgery in the Department of Plastic and Cosmetic Surgery at the Second Hospital of Hebei Medical University. The skin tissues were cut into pieces (3 mm × 3 mm × 2 mm), placed in complete Dulbecco’s Modified Eagle’s Medium (DMEM) containing 10% fetal bovine serum (FBS) (Gibco, Grand Island, NY, U.S.A.), 1% penicillin (100 U/ml), streptomycin (100 U/ml), and cultured in a 5% CO_2_ incubator at 37°C. Upon reaching 70–80% confluence, the HDFs were digested and passaged using trypsin/EDTA.

Cells cultured from the tissue blocks were characterized using immunohistochemistry with mouse anti-human vimentin monoclonal antibody and mouse anti-CK monoclonal antibody (dilution: 1:100, ZhongShan JinQiao, Beijing, China).

### Preparation of CGF

CGF was prepared from the venous blood of three healthy volunteers (age range: 24–30 years) who had provided informed consent before the experiment. The venous blood was directly extracted into a sterile 9 ml special anticoagulant vacuum tube and centrifuged vigorously for 13 min as per the following protocol: 2700 rpm for 2 min, 2400 rpm for 4 min, 2700 rpm for 4 min, and 3000 rpm for 3 min using a centrifuge (Medifuge, Silfradentsr, S. Sofia, Italy). The blood sample was found to separate into three layers; from top to bottom these layers included the red blood cell layer, CGF fibrin layer, and plasma layer [20]. The CGF liquid was then transferred into a fresh tube, sterilized using a 0.22-μm filter, and stored at −20°C.

### UVA irradiation and CGF treatment of HDFs

The radiation doses suitable for the experiment were first identified. HDFs were seeded into a 96-well plate at a density of 1 × 10^4^ cells per well in a volume of 200 μl. On the second day, the cells were washed using phosphate-buffered saline and exposed to different doses of UV radiation [0 (dark treatment), 5, 10, 20, and 30 J/cm^2^] at a radiation distance of 15 cm. A desktop instrument (Sigma Hightech, Shanghai, China) was used as the UVA light source. Before each experiment, the radiation intensity was measured using an UV radiation illuminant (Beijing Normal University Photoelectric Instrument Factory, Beijing). The 96-well plate was placed on ice during irradiation. The irradiated cells were added into complete culture medium and cultured at 37°C in an incubator containing 5% CO_2_. 3-(4,5-dimethyl-2-thiazolyl)-2,5-diphenyl-2-H-tetrazolium bromide (MTT) colorimetric assay was then performed on these cells.

We also determined the optimal concentration of CGF for treatment of the irradiated HDFs. Following irradiation with 20 J/cm^2^ UVA, the cells were cultured for 72 h in media containing different concentrations of CGF (5, 10, 15, and 20%). MTT colorimetric assay was performed every 24 h.

### Evaluation of cellular viability

The viability of HDFs following treatment with different concentrations of CGF was assessed using MTT assay. Twenty microliters of 5 mg/ml MTT was added to each well of the 96-well plates and the cells were cultured in an incubator containing 5% CO_2_ at 37°C. After 4 h, 150 ml DMSO was added to each well, followed by shaking for 10 min at room temperature. A microplate reader (Biotek, U.S.A.) was used to read the optical density of the plates at a wavelength of 570 nm.

### Experimental design

Three groups of cells were used in the present study: control group (without UVA radiation + DMEM with 10% FBS); UVA group (20 J/cm^2^ UVA radiation + DMEM with 10% FBS); and CGF group (20 J/cm^2^ UVA radiation + DMEM with 10% FBS + 20% CGF). The cellular morphology was observed under a microscope immediately after UVA irradiation as well as 24 h later.

### Intracellular ROS detection

HDFs were seeded into two 6-cm petridishes and cultured in complete culture medium. After 24 h, the respective treatments were given to the three groups of cells. Following that, DCFH-DA (1:1000, Beyotime, China) was added to the cells for 20 min and washed three times using DMEM. One of the plates was subjected to confocal laser scanning microscopy imaging (Leica SP8, Germany) at an excitation wavelength of 490 nm and an emission wavelength of 530 nm to observe the level of ROS fluorescence. The cells in the other dish were digested, centrifuged, and collected in the dark for flow cytometry analysis. Flow cytometry (excitation wavelength = 488 nm, emission wavelength = 525 nm, Beckman Coulter, America) was used to measure the fluorescence intensity of ROS in the three groups.

### Real-time polymerase chain reaction

Total RNA was extracted from the three groups of cells using TRIzol reagent (Invitrogen, U.S.A.), according to the manufacturer’s instructions. RNA concentration was measured using spectrophotometric readings at 260 and 280 nm. The RevertAid^™^ First Strand cDNA Synthesis kit (MBI Fermentation, ON, Canada) was used to reverse transcribe the extracted mRNA into cDNA, according to the manufacturer’s instructions. cDNA was amplified using a 7500 Real-time PCR System (Applied Biosystems, Foster City, CA, U.S.A.) and the mRNA levels of each group were normalized to the levels of GAPDH. The primer sequences used are as follows: MMP-1, forward 5′-TGG GCT GAA AGT GAC TGG GA-3′, reverse 5′-GGT CCA CAT CTG CTC TTG GC-3′; c-Jun, forward 5′-GCG GAC CTT ATG ACA GTA ACC-3′, reverse 5′-CCG TTG CTG GAC TGG ATT ATC AGG-3′; P38, forward 5′-GGC TCC TGA GAT CAT GCT GAA CTG-3′, reverse 5′-AGT CAA CAG CTC GGC CAT TAT GC-3′; GAPDH, forward 5′-TGC GCA CAA ATC CCT TCT-3′, reverse 5′-TTC AAG CCC ATT TGG CAG TT-3′. The expression levels of *MMP-1, c-Jun*, and *P38MAPK* were calculated using the 2^−ΔΔ*C*_T_^ method.

### Protein extraction and Western blot analysis

The expression levels of MMP-1, c-Jun, and P38 were detected using Western blot. Cells were lysed in PIPA buffer (Priole, China) to extract the whole protein. Thirty micrograms of total protein samples was mixed with 5× protein gel electrophoresis loading buffer and denatured at 95°C for 10 min. After gel electrophoresis, the separated protein bands were transferred to a polyvinylidene fluoride (PVDF) membrane, blocked using 5% nonfat dry milk at 37°C for 1 h, and then incubated with a working solution of antibody (MMP-1, c-Jun, and p38 1:1000, Santa Cruz Biotechnology, U.S.A.) at 4°C overnight. On the second day, the PVDF membrane was incubated with secondary anti-rabbit (1:2000, Bioeasy BE0101, China) at room temperature for 120 min. Digital science software (ImageJ) was used for grayscale analysis.

### Statistical analysis

SPSS 21.0 statistical software was used for statistical analysis. All data have been expressed as mean ± SE. Comparisons between multiple groups were carried out using one-way ANOVA or non-parametric tests. A *P-*value of <0.05 was considered statistically significant.

## Results

### Primary culture of HDFs

**Figure 1:**
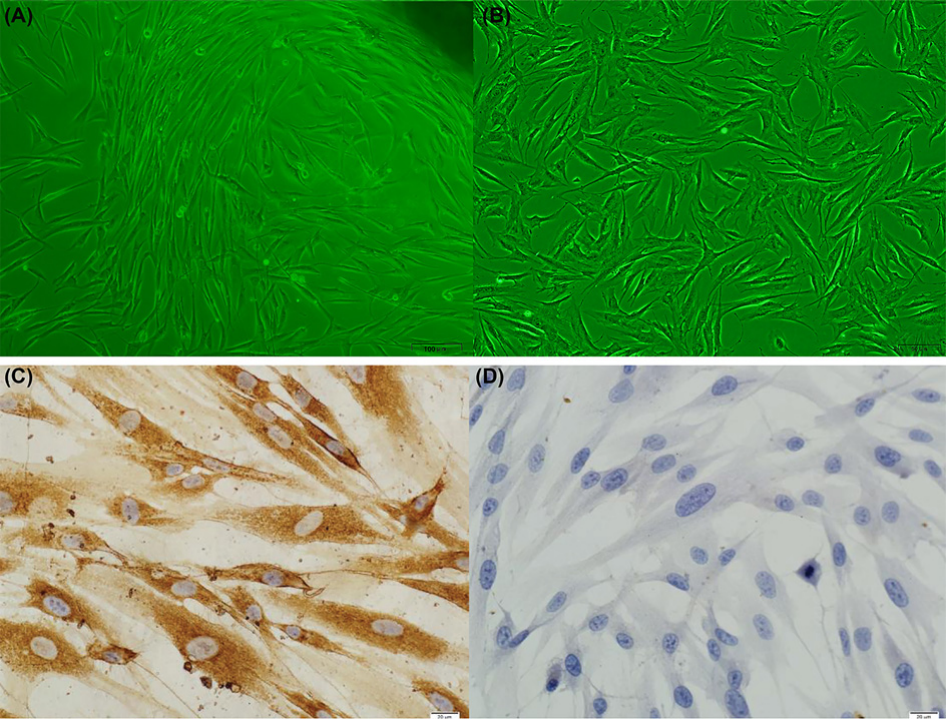
Primary culture of HDFs (**A**) Primary culture of HDFs (inverted microscope, 100×). (**B**) Subculture of HDFs (inverted microscope, 200×). (**C**) Representative images of vimentin^+^ HDFs (Polymer, 400×); and (**D**) CK-HDFs (polymer, 400×).

Five to seven days post establishment of primary culture of the skin tissue, a small number of fibroblast-like cells appeared out of the tissue pieces. After 4–5 days, these fibroblast-like cells grew radially around the tissue mass and fused into a monolayer (Figure 1A,B). When the fusion rate reached 70–80%, the cells were digested, centrifuged, and passaged. Immunocytochemistry staining showed that the fibroblast-like cells were vimentin positive and CK negative (Figure 1C,D).

### Morphology of HDFs in different treatments

**Figure 2:**
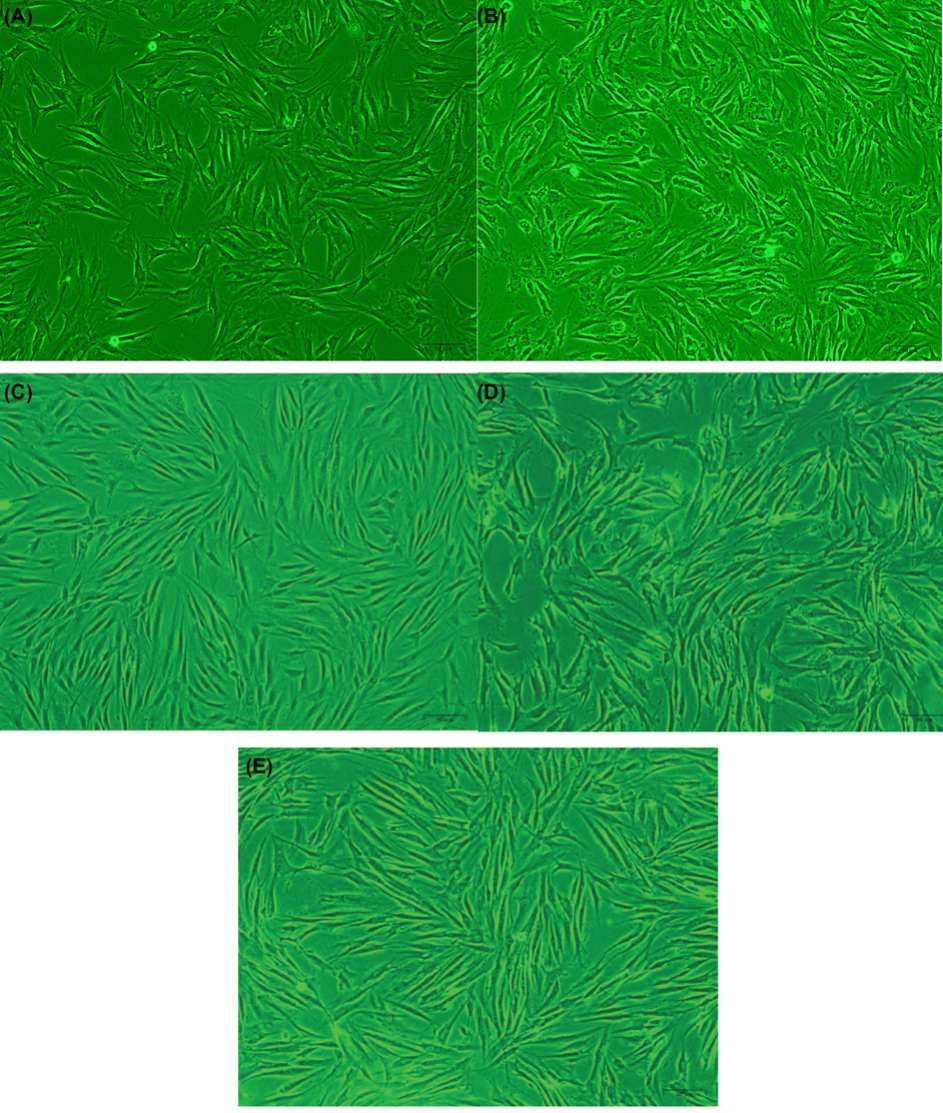
Morphological observation of HDFs in different treatment groups (**A**) Control group HDFs (200×); (**B**) control group HDFs cultured for 24 h (200×); (**C**) HDFs after 20 J/cm^2^ UVA exposure (200×); (**D**) UVA group HDFs cultured for 24 h (200×); and (**E**) CGF group HDFs cultured for 24 h (200×).

Control group: normal HDFs were spindle-like (Figure 2A); 24 h after complete culture, the cells grew larger, expanded, and increased in number (Figure 2C).

UVA group: Following irradiation, HDFs became swollen, rounded, atrophied, and ruptured, while a small number of cells were suspended (Figure 2B). Twenty-four hours after complete culture, the cell morphology became irregular and the number of cell fragments and suspended cells gradually increased (Figure 2D).

CGF group: Twenty-four hours after treatment with 20% CGF, the cell morphology was more regular than before CGF treatment; in addition, there was a reduction in the number of atrophied and suspended cells (Figure 2E).

### UVA dose screening

**Figure 3:**
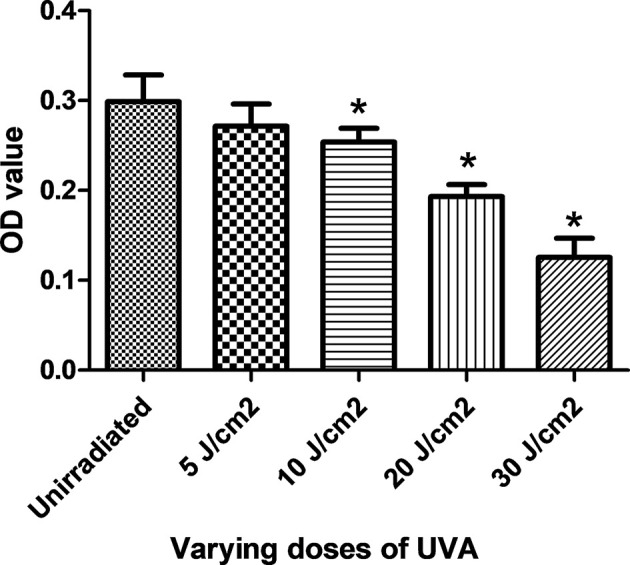
Cellular viability of HDFs following UVA radiation The OD values of HDFs after four different doses of UVA treatment, *P<0.05 as compared with unirradiated group.

MTT results showed that the cell survival rate decreased in a dose-dependent manner under different irradiation intensities. The HDF activity under 20 J/cm^2^ UVA irradiation was close to the half the lethal rate (Table 1); thus, it was used as the test UVA irradiation dose in the photoaging model of the subsequent experiment. The cell viabilities of the 10, 20, and 30 J/cm^2^ irradiation groups were significantly lower (*P*<0.05) than that of the control group (Figure 3). However, the 5 J/cm^2^ irradiation group was not significantly distinguishable from the control group (*P*>0.05) (Figure 3).

**Table 1:** Absorbance values of HDFs at different irradiation doses 
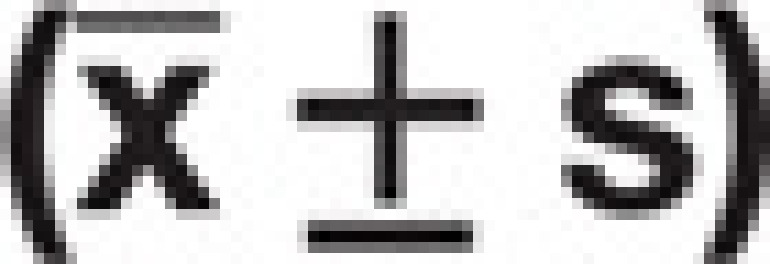

Group	OD value	Survival rate
Control	0.299 ± 0.030	
5 J/cm^2^	0.272 ± 0.025	91%
10 J/cm^2^	0.254 ± 0.015*	84%
20 J/cm^2^	0.193 ± 0.013*	64%
30 J/cm^2^	0.125 ± 0.021*	41%

* *P*<0.05, compared with the control.

### Identification of the working concentration for CGF

**Figure 4:**
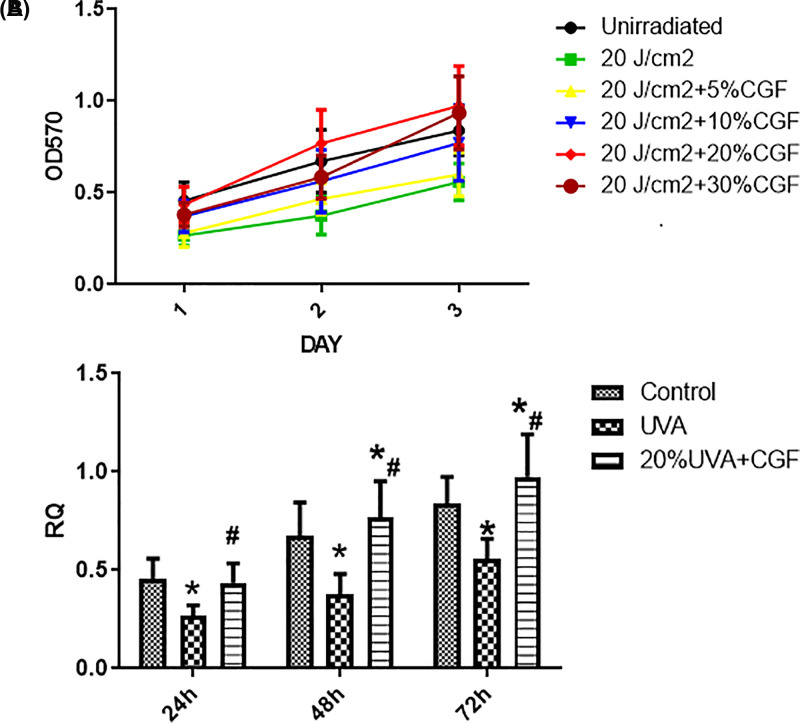
Cellular viability of HDFs following UVA radiation combined with CGF treatment (**A**) Growth curves for six groups of cells. (**B**) The OD value of three groups of HDFs cultured for 72 h. *P<0.05 compared with control group; ^#^*P*<0.05 compared with UVA group.

As shown in Figure 4A,B, the CGF-treated cells showed higher cell viability than the UVA-treated cells. Cells treated with 20% CGF had the highest survival rate among the control, UVA alone, 5, 10, and 30% CGF treated-groups. Therefore, we used 20% CGF as the optimal dose in the present study.

### CGF treatment reduced the intracellular ROS levels

**Figure 5:**
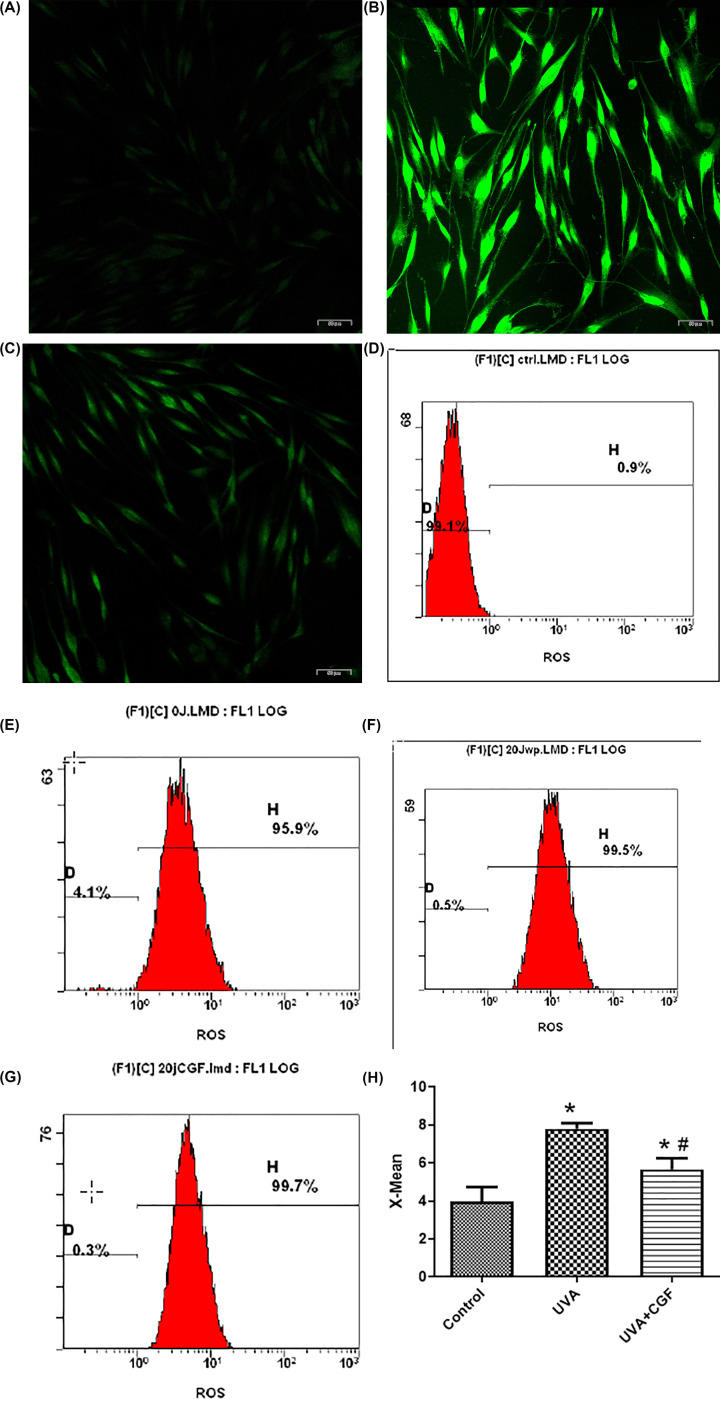
ROS induction of HDFs following UVA radiation combined with CGS treatment (**A**) Green fluorescent expression of ROS in the control group HDFs (200×). (**B**) Green fluorescent expression of ROS in the UVA group HDFs (200×). (**C**) Green fluorescent expression of ROS in the CGF group HDFs (200×). (**D**) Flow cytometry results of negative control group (untreated HDFs without DCFH-DA probe) control group. (**E**) Flow cytometry results of control group. (**F**) Flow cytometry results of UVA group. (**G**) Flow cytometry results of UVA+CGF group. (**H**) The X-mean of three group HDFs, *P<0.05, compared with the control group, ^#^*P*<0.05, compared with UVA group.

Following DCFH-DA staining, the ROS in the cells were detected in the form of green fluorescence. Compared with the control group, the UVA group showed higher green fluorescence; CGF treatment was found to rescue the fluorescence increase caused by UVA (Figure 5A–C).

The average fluorescence intensity in the three groups was detected using flow cytometry, as shown in Figure 5D–G. The average fluorescence intensity of cells in the UVA group was significantly higher than that in the control group (*P*<0.05). Relative to the UVA group, CGF treatment significantly reduced the formation of ROS (*P*<0.05) (Figure 5H).

### CGF decreased the expression levels of P38, c-Jun, and MMP-1 in HDFs

**Figure 6:**
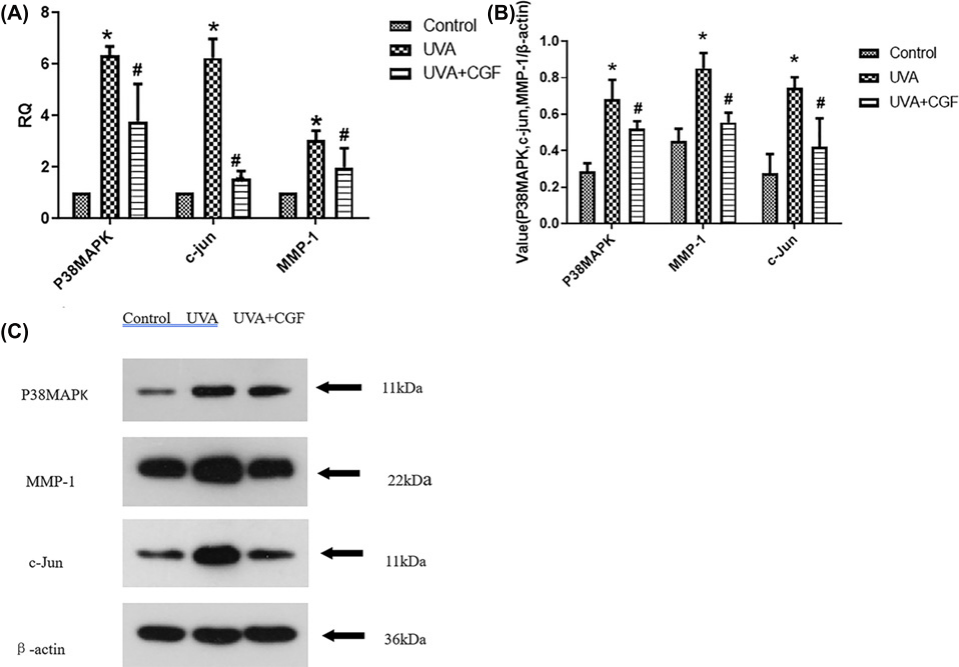
Expression levels of P38MAPK, c-Jun, and MMP-1 in HDFs (**A**) mRNA expression levels of P38MAPK, c-jun, and MMP-1 in HDFs. *P<0.05, compared with the control group, ^#^*P*<0.05, compared with UVA group. (**B**) Western blotting of the P38MAPK, c-jun, MMP-1 protein in the three groups. (**C**) Expression of the P38MAPK, c-jun, and MMP-1 protein levels in the three groups. *P<0.05, compared with the control group, ^#^*P*<0.05, compared with UVA group.

Results of the RT-PCR assay showed that the mRNA levels of MMP-1, c-Jun, and P38 were markedly elevated in the cells from the UVA group compared with the control group (*P*<0.05). Upon 20% CGF treatment, a significant decrease was seen in the UVA-induced MMP-1, c-Jun, and P38 mRNA expression levels (*P*<0.05) (Figure 6A).

The protein expression levels, as detected by Western blotting analysis are shownin Figure 6B. The levels of MMP-1, c-Jun, and p38MAPK proteins were statistically higher in the UVA group, compared with the control group; however, the levels of MMP-1, c-Jun, and p38MAPK proteins were significantly lower in the CGF-treated group, compared with the UVA group (Figure 6C, *P*<0.05).

## Discussion

HDFs are the most important cell components in the dermis. They produce collagen, fibronectin, glycosaminoglycan, and other extracellular matrix, which support the whole dermis structure and maintain skin elasticity and moisture. UVA radiation is a strong type of UV radiation that can damage HDFs, resulting in the reduction in fibroblasts, abnormal secretion/synthesis function, and skin aging [21,22]. Skin in the face and neck regions is most prone to radiation-induced aging because of less shielding and longer direct exposure time to sunlight as compared with other parts of the skin. Keeping the same in mind, we selected human eyelid tissue for HDF primary culture. There is no gold standard for the selection of the indoor and outdoor UVA experimental dose. Some studies have used a cell survival rate of 50% as the selection criterion for radiation dose [23]. Following literature and a previous study from our laboratory [19], we used 5, 10, 20, and 30 J/cm^2^ of UVA to irradiate HDFs and screen the appropriate UVA irradiation dose. The results showed that the 24 h survival rate following irradiation with 20 J/cm^2^ UVA was 64%, which met the requirements of the present study and thus was used as the UVA irradiation dose for the following experiment.

At present, many antioxidants, including red raspberry extract, neferine, resveratrol, lycopene, etc. [24] have been shown to have positive effects on the cell damage caused by UV radiation; but these antioxidants present some disadvantages as well, such as allergy, low rate of absorption, and utilization. As a new generation of platelet concentrate, CGF has superior potential in terms of clinical and biotechnological applications. CGF eliminates the risk of infection and immune rejection. At present, no side effects have been reported for CGF application. Special centrifugation speed can activate platelets and increase the frequency of collision and rupture between platelets; this induces the platelets to release many growth factors and cytokines. In the process of platelet activation, growth factors in CGF are released slowly, ensuring that the concentration of growth factors is maintained at a certain level for a period of time (8–10 days), thus contributing to the repair of damaged tissues [25,26]. It has been reported that direct injection of CGF into the face can smoothen wrinkles for a long time without side effects such as infection, scar, pigmentation, and inflammation [27]. At present, no side effects of CGF have been reported in clinical application. The plausible reason may be that the CGF is derived from autogenous venous blood, thereby eliminating the risk of immune rejection. In this experiment, liquid CGF was prepared using centrifugation and most of the fibrin was removed using a 0.22-μm filter. It was necessary to screen the working concentration of CGF to be used in this experiment and our results showed that 20% CGF had the best effect on cell viability.

Based on a previous finding that CGF can inhibit UVA-induced photoaging damage caused to the HDFs [19], this study further explored the possible mechanism for the same. UV radiation-induced photoaging begins with oxidative damage caused by the cellular response to external stimuli. ROS is a general term for superoxide anion (O^2−^), hydrogen peroxide (H_2_O_2_), hydroxyl radical (OH^−^), ozone (O^3^), and singlet oxygen (^1^O_2_) produced by the mitochondrial inner membrane. Under normal circumstances, the body’s antioxidant system can remove the excessive ROS produced by cells. Oxidative stress occurs when the production of ROS exceeds the scavenging capacity of the antioxidant system [28]. Excessive ROS can affect the structure and function of HDFs, destroy the structure of lipids, proteins, and nucleic acids, and participate in cell apoptosis, photoaging, immunosuppression, and photocarcinogenesis [29,30]. Therefore, inhibiting or removing ROS can reduce the occurrence of oxidative damage. The results of the present study show that CGF plays an antioxidative role by reducing the UVA radiation-induced production of ROS in HDFs.

ROS is a secondary messenger in signaling pathways and gene expression regulation. It can activate MAPK/AP-1 pathways and mediate ECM degradation [9]. MAPK is an important transmitter of signals from the cell surface to the nucleus [31]. It receives signals from membrane receptors and transports them into the nucleus, thus participating in cell growth, proliferation, differentiation, and other signaling pathways. P38 is a subclass of MAPK protein kinases. UV radiation can induce phosphorylation of P38MAPK; phosphorylated P38MAPK can subsequently trigger the transcription of c-Jun [32]. The expression of c-Jun is regulated by UV radiation, while the constitutive expression of c-Fos in human skin is not further induced by UV radiation. Under UV irradiation, c-Fos and c-Jun heterodimers are induced to form the transcription factor AP-1 [33,34], which continues to activate MMPs. MMPs participate in the degradation of various components of the ECM proteins [35]. Type I and III collagen fibers are the most abundant collagen fibers in the skin, while the main components of elastic fibers are type III collagen fibers. MMP-1 can degrade these two kinds of collagen fibers and reconstitute the ECM, resulting in wrinkles, sagging, and other signs of skin aging [36,37]. The results of the present study showed that the expression of P38MAPK, c-Jun, and MMP-1 was lower in the UVA+CGF group than in the UVA group. The results showed that CGF reduced the production of ROS in cells, inhibited activation of the P38MAPK/AP-1 signaling pathway, and reduced the expression of MMP-1, therefore inhibiting the photoaging of HDFs.

## Conclusions

In summary, CGF protected the HDFs against UVA radiation; CGF mediated this effect through a reduction in the ROS accumulation in cells and an inhibition of the MAPK pathway activation, thus preventing the degradation of dermal fibers. The mechanism of action proposed in the present study provides a theoretical basis for the early application of CGF in clinical practice.

## Abbreviations

BMK1: big mitogen-activated Protein kinase 1
CGF: concentrated growth factor
DCFH-DA: dichloro-dihydro-fluorescein diacetate
DMEM: Dulbecco’s modified eagle’s medium
ERK: extracellular regulated protein kinases
ECM: extracellular matrix
FBS: fetal bovine serum
GAPDH: glyceraldehyde-3-phosphate dehydrogenase
HDF: human dermal fibroblast
MMP: matrix metalloproteinase
PVDF: polyvinylidene fluoride
P38MAPK/AP-1: P38 mitogen-activated protein kinase/activated protein-1
ROS: reactive oxygen species
UV: ultraviolet
